# Born to fail: flaws in replication design produce intended results

**DOI:** 10.1186/s12916-020-01517-w

**Published:** 2020-03-26

**Authors:** Abraham D. Flaxman, Riley Hazard, Ian Riley, Alan D. Lopez, Christopher J. L. Murray

**Affiliations:** 1 Institute for Health Metrics and Evaluation, University of Washington, 2301 5th Avenue Suite 600, Seattle, WA 98121 USA; 2 Melbourne School of Population and Global Health, The University of Melbourne, Carlton, VIC Australia

**Keywords:** Verbal autopsy, Reproducible research, InSilicoVA, Tariff

## Abstract

We recently published in *BMC Medicine* an evaluation of the comparative diagnostic performance of InSilicoVA, a software to map the underlying causes of death from verbal autopsy interviews. The developers of this software claim to have failed to replicate our results and appear to have also failed to locate our replication archive for this work. In this Correspondence, we provide feedback on how this might have been done more usefully and offer some suggestions to improve future attempts at reproducible research. We also offer an alternative interpretation of the results presented by Li et al., namely that, out of 100 verbal autopsy interviews, InSilicoVA will, at best, correctly identify the underlying cause of death in 40 cases and incorrectly in 60 – a markedly inferior performance to alternative existing approaches.

## Background

We welcome the effort that Li et al. [1] have put into replicating the findings of our recent paper [2], demonstrating the comparatively modest performance of the InSilicoVA algorithm in diagnosing the cause of death from verbal autopsy. In our analysis, we found that chance corrected concordance (CCC) for their approach varied from 16.1 to 38.8, compared with 37.8 to 52.5 for Tariff 2.0 [3]; furthermore, we found that the chance corrected cause-specific mortality fraction (CSMF), arguably the most important metric of diagnostic performance for informing public policy debates, varied from an astonishingly poor -59.4 to 37.6. In other words, policy-makers could expect that the method would, at best, correctly predict cause of death patterns in a population less than 40% of the time. Having said that, we firmly believe that reproducibility is the hallmark of the scientific method and we hold transparency and open science as fundamental values.

### Replication archives and where to find them

Nevertheless, there is one major claim made by Li et al. [1] with which we fundamentally disagree. They argue that we have not released a replication archive for our work, yet it can be readily found online [4]. Had Li et al. [1] identified this publicly available, open-source Python code, it could have substantially facilitated their replication attempt, making their work both easier and more accurate. We are unaware of why the authors chose not to apply our replication archive, but it could be that, without an explicit reference in a manuscript, many will not think to search for a replication archive on their own. We therefore suggest that, for future replication efforts, authors should generate document object identifiers (DOIs) for replication archives and that these should be included in the reference section of scholarly communications such as journal articles; journals could even require this practice, which involves quite a simple operation. An additional benefit of this approach may be in the preservation of research archives – GitHub and other commercial services for distributed concurrent version control are undoubtedly useful for reproducible computational research, but they are not designed to be scholarly archives and should not be relied upon as such. At the time of writing, it is relatively simple to generate an archival version of a specific version of a Git repository using Zenodo, which we have now done for our original research archive [5].

### Replication or reproduction?

There has been an unfortunate conflict in the terminology used to describe reproducible research in recent years; some researchers have applied a very specific meaning of replication and a different meaning for reproduction, while others have used these same terms with exactly opposite meanings [6]. Li et al. [1] never clearly state what sort of replication they are attempting – do they search for “*consistent results across studies aimed at answering the same scientific question, each of which has obtained its own data*”? (this is the emerging consensus definition of replicability, as codified in a recent National Academy report [7]). Since they have relied on the same database of gold-standard verbal autopsy interviews as we used in our study, it seems more likely that they are attempting a ‘reproduction’ in the sense of reproducible computational research, seeking to obtain “*consistent computational results using the same input data, computational steps, methods, code, and conditions of analysis*”, such as done by Donoho [8]. Yet, while the authors have applied an updated version of their InSilicoVA algorithm in their work, they have selectively focused on only one of the nine scenarios that we analyzed, thereby violating the requirement to use the same code/conditions of analysis.

We offer an alternative conclusion from a review of their findings, namely that they have succeeded in a (albeit limited) replication of one of our results – that is, for every 100 deaths, InSilicoVA will predict the correct cause in 30 to 40 cases only. At the population level, this implies a chance-corrected CSMF Accuracy inferior to the published metrics for Tariff 2.0 in settings where the deceased had access to healthcare (a ‘healthcare experience’) [3]. On the other hand, their results do show an improved chance-corrected CSMF Accuracy in cases where a ‘healthcare experience’ did not apply, possibly reflecting an improvement in more recent versions of their algorithm. InSilicoVA is now of similar diagnostic accuracy to Tariff 2.0, in at least one setting, that is for adult deaths in a community with limited healthcare access.

### Room for improvement

We suggest some areas where the replication analysis of Li et al. [1] could be improved. First, instead of using the standard approach of a stratified train–test split [9], Li et al. [1] have divided the training and test sets without ensuring that all causes are represented in the testing set. This makes it impossible for them to realize the resampling required by the CSMF they draw from – the Dirichlet distribution. In lines 37–42 of their codes/fit. R script, they address this in a way that produces a distribution that is not the Uninformative Dirichlet that has been previously used for measuring CCC and CSMF Accuracy. The magnitude of the error introduced by this choice is unclear but may well lead to a substantial overestimation of CCC and CSMF Accuracy for their method.

Similarly, the uncertainty intervals reported by Li et al. [1] are not appropriate for comparison with previously published results [2, 3, 10, 11]. We have defined CCC and CSMF Accuracy as the median across all cause fractions, and we have therefore always reported the 95% confidence interval of the median to quantify uncertainty. The wide confidence intervals published by Li et al. [1] suggest, and their replication archive confirms, that they have published the spread of the distribution for their uncertainty interval. This is a valid construct but it should not be compared with previously published values, and we find it less relevant than a quantification of the uncertainty in the median – our uncertainty interval can be used as an informal significance test to determine if the superior median of one method or setting appears better than another simply due to chance.

## Conclusion

One interpretation of Li et al.’s [1] final paragraph is a proposal that methodological innovation in global health should be vetted by the American Statistical Association through publication in that organization’s quarterly journal – we whole-heartedly reject this as a principle. We believe that other open access journals, like *BMC Medicine*, have an important role to play in the scientific review and public health implications of methodological innovation. This leaves unanswered the question of who is responsible for vetting the claims of published results; we believe that this dilemma has no easy answer, although emerging best practices in reproducible computational research provide some steps forward. Clearly, a journal’s peer-review process must ensure rigorous statistical review of the methods used – to leave this to authors would be analogous to allowing the tobacco industry to handle their own regulations. On the other hand, it is essential that published results be both replicable and reproducible, as this is one of the key elements of the scientific method. Science must not rely on the authority of organizations (be it the American Statistical Association or any other) but rather on logic and evidence. To reiterate one practice from computational reproducible research that can facilitate this, in addition to publishing source code as a replication archive, we suggest that authors create an archived version of this code with a DOI.

There is one additional mechanism that scholarly publishing can offer for future efforts to conduct replication studies – pre-registration. Had Li et al. [1] shared an analysis plan, including their criteria for what they considered to be ‘replication’, we could have pointed them to the source code we had published. We could also have discussed, prior to their publication, our alternative interpretation of their results, namely that their work has in fact reinforced our findings in at least one of the nine cases reanalyzed, i.e., that out of 100 autopsy interviews, InSilicoVA will, at best, correctly identify the underlying cause of death in 40 cases and incorrectly in 60.

## Data Availability

Not applicable.
